# Genome-wide modeling of complex phenotypes in *Caenorhabditis elegans* and *Drosophila melanogaster*

**DOI:** 10.1186/1471-2164-14-580

**Published:** 2013-08-28

**Authors:** Supriyo De, Yongqing Zhang, Catherine A Wolkow, Sige Zou, Ilya Goldberg, Kevin G Becker

**Affiliations:** 1 Gene Expression and Genomics Unit, Laboratory of Genetics, National Institute on Aging, National Institutes of Health, Biomedical Research Center, 251 Bayview Boulevard, Baltimore, MD 21224, USA; 2 Department of Neuroscience, Albert Einstein College of Medicine, Bronx, NY 10461, USA; 3 Translational Gerontology Branch, National Institute on Aging, National Institutes of Health, Biomedical Research Center, 251 Bayview Boulevard, Baltimore, MD 21224, USA; 4 Image Informatics and Computational Biology Unit, Laboratory of Genetics, National Institute on Aging, National Institutes of Health, Biomedical Research Center, 251 Bayview Boulevard, Baltimore, MD 21224, USA

**Keywords:** *C*. *elegans*, *D*. *melanogaster*, Worm, Fly, Aging, Gene set, Phenotype, Ontology, Network, Gene expression

## Abstract

**Background:**

The genetic and molecular basis for many intermediate and end stage phenotypes in model systems such as *C*. *elegans* and *D*. *melanogaster* has long been known to involve pleiotropic effects and complex multigenic interactions. Gene sets are groups of genes that contribute to multiple biological or molecular phenomena. They have been used in the analysis of large molecular datasets such as microarray data, Next Generation sequencing, and other genomic datasets to reveal pleiotropic and multigenic contributions to phenotypic outcomes. Many model systems lack species specific organized phenotype based gene sets to enable high throughput analysis of large molecular datasets.

**Results and discussion:**

Here, we describe two novel collections of gene sets in *C*. *elegans* and *D*. *melanogaster* that are based exclusively on genetically determined phenotypes and use a controlled phenotypic ontology. We use these collections to build genome-wide models of thousands of defined phenotypes in both model species. In addition, we demonstrate the utility of these gene sets in systems analysis and in analysis of gene expression-based molecular datasets and show how they are useful in analysis of genomic datasets connecting multigenic gene inputs to complex phenotypes.

**Conclusions:**

Phenotypic based gene sets in both *C*. *elegans* and *D*. *melanogaster* are developed, characterized, and shown to be useful in the analysis of large scale species-specific genomic datasets. These phenotypic gene set collections will contribute to the understanding of complex phenotypic outcomes in these model systems.

## Background

Traditional experimentation in animal model systems such as the worm Caenorhabditis elegans and the fly Drosophila melanogaster often results in complex molecular and phenotypic outcomes. Frequently a targeted deletion or ectopic expression of a single gene product results in pleiotropic phenotypes. Similarly, broad high-throughput multiplex experimental strategies such as microarray based gene expression, RNA interference (RNAi) screens, or next-generation DNA and RNA sequencing, analyzing phenomena such as development, behavior, mating, diet, and life span, typically produce large datasets requiring complex analytical approaches.

Gene sets are collections of keyword terms with annotated genes derived from multiple sources of a priori information. They have been used in computational analysis of gene expression data [1-3] with the goal of identifying higher order relationships beyond simple gene list results, as well as in analysis of population based GWAS in humans [4,5]. The most commonly used gene sets include those derived from GO annotations [6], biological pathways from KEGG [7] or BioCarta, expression modules, DNA binding sites, or other sources of molecular information [1,3,8]. Each collection of gene sets has its own unique qualities and features which are useful in different ways. For instance, KEGG emphasizes metabolic and biochemical pathways; GO annotations, while having some phenotypic content, emphasizes molecular function, cellular component, and biological processes, while MSigDB [8] emphasizes gene expression signatures. This information is often closely related, or “proximal” to gene and molecular function, rather than more “distal” information regarding phenotypic outcomes and disease susceptibility. Recently, phenotype based gene sets have been derived exclusively from genetically determined phenotypic associations for mouse phenotypes and common human disease [9,10], resulting in gene sets for specific phenotypes, organized by a structured systematic ontology.

Here, we present gene sets for worm and fly, which use the structured ontology found in the Worm Phenotype Ontology from the *C*. *elegans* database - WormBase [11] and phenotypic descriptions for *D*. *melanogaster* found in FlyBase [12]. These gene sets are derived from information on gene-phenotype relationships based on genetically determined phenotypes. We use these collections in large scale phenotypic modeling in worms and flies and demonstrate their utility in complex analysis in multiple ways, including analysis of gene expression datasets representing complex phenotypic and biological phenomena in both *C*. *elegans* and *D*. *melanogaster*. In this way, we integrate large scale genome analysis with large scale phenotypic analysis in these two model systems.

## Results

### Derivation of worm gene sets

The worm gene sets presented here are derived from two lists of genes and assigned phenotypes provided by Gary Schindelman and Paul Sternberg as a component of the Worm Phenotype Ontology [13]. These two lists originated from information curated from RNAi experiments and genetic variations (VAR) as archived in WormBase [14].

Two worm gene set files (CE- RNAi-GS and CE-VAR-GS) were produced by parsing each gene list separately into non-redundant lists of unique phenotypic terms with all genes assigned to their corresponding phenotypic terms. This produced two non-redundant gene set files containing 850 and 1109 gene sets for RNAi and VAR, respectively. In addition, we developed a master worm file by combining the original RNAi and VAR gene lists into a combined file (CE-Combined-GS) containing 1,385 non-redundant phenotypes and their associated gene sets.

### Derivation of fly gene sets

The *Drosophila* gene sets described here are derived from phenotypic data provided in FlyBase (see Methods). A file containing 259,162 phenotypic descriptions with assigned *Drosophila* genes was collapsed and parsed resulting in a non-redundant gene set file of 11,999 unique phenotypic terms with annotated genes. This file named DM-narrow-GS was used for systems biology and gene expression analysis.

Table 1 shows representative examples of individual gene sets from the *C*. *elegans* and *D*. *melanogaster* gene set files. Official gene symbols are shown where available, locus tags (*C*. *elegans*) where gene symbols are not available. As in other gene set collections, as the number of genes in any given gene set decreases, the phenotypes progress from broad categories to more specific phenotypic descriptors. The full gene set lists consist of a wide range of developmental, structural, metabolic and behavioral phenotypes, representing a large majority of the experimentally determined phenotypes found in worms and flies. They range from broad phenotype categories such as “sterile”, “slow_growth”, or “larval_arrest” in worms and “viable”, “lethal” and “fertile” in flies; to narrow phenotypic descriptors such as “flaccid”, “DNA_synthesis_variant” or “no_posterior_pharynx” in worms and “ejaculatory_bulb”, “dorsal_vessel_primordium”, or “dense_body” in flies. In addition, there is often overlap of the genes found in related gene sets in both species, emphasizing the contributions of the same genes to multiple phenotypic traits. The complete *C*. *elegans* (Additional file 1: Table S1: Additional file 2: Table S2: Additional file 3: Table S3) and *D*. *melanogaster* (Additional file 4: Table S4) gene set files are available at this address http://www.grc.nia.nih.gov/branches/rrb/dna/index/Worm-fly_gene_sets_5-9-12.html.

**Table 1:** Selected Phenotype gene sets

***C. elegans***		
**Large gene sets**	**Number**	**Genes**
embryonic lethal	3301	AC7.1(tag-49), AC7.10, AC7.13, AC7.19, AC7.68, AC8.6, AH6.5, B0001.2, B0025.2 9(csn.2), B0025.5, etc…
larval arrest	1688	AC8.6, B0025.1(vps-34), B0035.10(his-45), B0035.11, B0035.12, B0035.7(his-47), B0035.8(his-48), B0205.6, B0238.11, B0250.1, etc…
slow growth	1664	AC3.7, AC8.6, B0024.4, B0025.1(vps-34), B0025.2, B0025.5, B0025.6, B0035.10(his-45), B0035.11, B0035.12, etc…
locomotion variant	1414	AH6.5 (mex-6), B0025.1(vps-34), B0035.11, B0035.12, B0035.14(dn-j1), B0035.15, B0035.7(his-47), B0035.8(his-48), B0035.9(his-46), B0207.4(air-2), etc…
maternal sterile	1107	AC7.1(tag-49), AC7.10, AC7.13, AC7.19, AC7.68, AC8.6, B0024.14, B0025.2, B0025.5, B0035.10(his-45), etc…
reduced brood size	975	AC8.1, AC8.2, B0035.10(his-45), B0035.11, B0041.4(rpl-4), B0205.6, B0207.4(air-2), B0252.9, B0261.2(let-363), B0261.4, etc…
sterile	889	B0035.11, B0035.8(HIS-48), B0041.4(RPL-4), B0205.6, B0212.4, B0284.1, B0284.6, B0285.1, B0286.4(NTL-2), B0304.1(HLH-1), etc…
larval lethal	834	AC7.11, AC7.15, AC7.18, AC7.2(soc-2), AC7.22, AC7.29, AC7.33, AC7.4, AC7.6, AC7.65, etc…
**Intermediate gene sets**		
cytokinesis fails early emb	57	B0207.4(air-2), B0273.2(puf-7), C01F6.3, C03C10.3(rnr-2), C07H6.5(cgh-1), C08B11.1(zyzg-11), C09G4.3(cks-1), C17G10.4(CDC-14), C25A1.9(rsa-1), C32E8.8(ptr-2), etc…
cell cycle slow early emb	56	C03C10.3(rnr-2), C08B11.1(zyzg-11), C14B9.4(plk-1), C26D10.2(hel-1), C27A2.3(ify-1), C30C11.2(rpn-3), C33H5.15(sgo-1), C40H5.6, C40H5.8, C47E12.5(uba-1), etc…
pharyngeal pumping reduced	49	B0348.4(egl-8), B0365.3(eat-6), B0412.2(daf-7), B0495.4(nhx-2), C02C6.1(dyn-1), C05D2.1(daf-4), C09B7.1(ser-7), C09B7.10, C09B7.12, C09B7.9, etc…
pronuclear size defective early emb	43	B0035.12, C08B11.1(zyzg-11), C08B6.9, C26D10.1(ran-3), C27A2.3(ify-1), C28C12.2, C37A2.4(cye-1), C38D4.3(mel-28), C40H5.6, C40H5.8, etc…
bag of worms	38	B0348.4(egl-8), B0412.2(daf-7), C04A2.3(egl-27), C04G2.7(egl-38), C05D9.5(ife-4), C08C3.1(egl-5), C26E6.8(ula-1), C30A5.7(uno-86), C44B12.2(ost-1), C46F4.1, etc…
exaggerated asynchrony early emb	36	C03C10.3(rnr-2), C25D7.6(mcm-3), C26D10.1(ran-3), C28C12.2, C29A12.3(lig-1), C38D4.3(mel-28), C39E9.13(rfc-3), C40H5.6, C40H5.8, C54G10.2(rfc-1), etc…
organism osmotic stress response var.	34	B0218.3(pmk-1), C12C8.1(hsp-70), C32E12.3(osr-1), C53B4.12, C53D6.18, F07C6.7, F10D2.9(fat-7), F11C7.5(osm-11), F19H8.1(tps-2), F38E11.1(hsp-12.3), etc…
dead eggs laid	27	C09D4.5(rpl-19), C27A2.2(rpl-22), C36E8.5(tbb-2), C47B2.3(tba-2), C53A5.1(ril-1), C54C6.2(ben-1), F25B5.4(ubq-1), F26D10.3(hsp-1), F26E4.8(tba-1), F44F4.11(tba-4), etc…
**Small gene sets**		
neuron function reduced	8	F36F2.5(tax-2), F55A8.2(egl-4), K03A11.3(ceh-28), K03A11.8, ZC416.8(unc-17), ZC84.2(tax-4), ZK1290.18, ZK1290.2(tph-1)
neuron morphology variant	8	C10A4.8(mnm-2), C35C5.4(mig-2), C44B11.3(mec-12), F28D1.10(gex-3), K10G9.3(pad-2), T01E8.4, Y51H4A.3, ZK154.3(mec-7)
pheromone induced dauer form. enhan	7	C38C3.5(unc-60), F02E8.6(ncr-1), F02E8.9, F55A8.2(egl-4), T20B5.3(oga-1), Y44A6D.4(sdf-9), Y6B3B.11(hsd-1)
programmed cell death variant	7	C07H6.7(lin-39), C09G4.1(hyl-1), F31E3.1(ceh-20), T07C4.8(ced-9), T12F5.4(lin-59), T28F12.2(unc-62), Y6B3B.10(lag-1)
cell division slow	6	C26D10.1(ran-3), C29E4.3(ran-2), F26B1.3(ima-2), F28B3.8(imb-1), K01G5.4(ran-1), ZK328.5(npp-10)
ectopic neurite outgrowth	6	B0285.5(hse-5), C35C5.4(mig-2), C39F7.2, F41C6.1(unc-6), T19B4.7(unc-40), T24B8.6(hlh-3)
dauer cuticle variant	5	C47G2.1(cut-1), C47G2.15, F22B5.3(cut-3), M142.2(cut-6), ZC328.1
endosome biogenesis variant	5	F49E7.1(rme-6), F58G6.1(amph-1), W06B4.3(vps-18), Y39A1A.5(rabx-5), Y49E10.11(tat-1)
***D. melanogaster***		
**Large gene sets**	**Number**	**Genes**
macrochaeta	927	arc, abb, abr, ac, Act5C, ade2, ade3, amb, aop, Appl, etc…
male sterile	854	abd-A, Abd-B, abt, ac, ade2, amb, ano, aop, ap, ar, etc…
wing vein	768	abd-A, Abd-B, abt, abw, ac, ade2, ade3, al, aop, ap, etc…
pigment cell	708	ade2, ade3, amb, aop, arm, bi, bo, bos, br, brb, etc…
ommatidium	704	a, Abd-B, Abl, abr, abt, ald, amx, aop, apx, arm, etc…
neurophysiology defective	404	Abl, Ace, acj6, Appl, Arr2, bas, baz, bi, bsk, bss, etc…
short lived	235	ap, cad, car, Cat, cm, comt, dnc, dor, ecd, EcR, etc…
body color defective	229	Abd-B, abt, amb, asx, b, bi, Bkd, br, cal, crm, etc…
**Intermediate gene sets**		
hyperplasia	67	arm, bam, cg, cos, Dl, ds, eyg, gd, ft, ImpL2, etc…
size defective	67	aop, arm, bi, dpp, ds, ena, ft, gt, Hsc70-4, L, etc…
learning defective	62	agn, cab, cbd, ccb, ccd, Ddc, dnc, eag, Fas2, G-salpha60A, etc…
increased cell size	55	brm, fkh, Hsc70-4, swm, mod, phl, Ras85D, shi, stg, Egfr, etc…
wing sensillum	54	arm, arr, ase, bi, brm, clm, Dr, dsh, eg, fu, etc…
scutum	53	abd-A, Abd-B, ac, ap, Bx, Pka-C1, Dl, dpp, Dr, ds, etc…
cell death defective	51	DNaseII, dor, dsh, EcR, dco, numb, Ras85D, rst, W, Top1, etc…
large body	44	ImpL2, l(2)gl, phl, Ras85D, rl, tkd, tor, tsh, gig, CycD, etc…
**Small gene sets**		
optic chiasm	8	bi, rst, so, sim, ato, tutl, Scer\GAL4, elav
CNS glial cell	7	E(z), sws, gcm, Scer\GAL4, spdo, hkb, vnd
adult myoblast	7	slou, N, Rac1, insc, Scer\GAL4, Hsap\CDKN1A, tw
sex comb tooth	7	Pc, Scr, ph-p, Scer\GAL4, KG01932, 5-SZ-3716, Zzzz\Aobl-tra
abdominal 3 seg. border muscle	6	if, numb, mys, insc, Tig, Scer\GAL4
epidermal cell	6	ed, pim, tkv, ct, Scer\GAL4, exo84
glomerulus	6	dnc, Wnt5, Cdc42, Scer\GAL4, drl, Drl-2
mesothoracic cleft	6	bsk, kay, pnr, Scer\GAL4, park, puc

### General uses of phenotype based gene sets in both worm and fly

As described here, a single gene set is essentially a single phenotypic term followed by a single row of genes that have been associated with that phenotype. A collection of gene sets consists of a list of phenotypic terms with their corresponding gene sets. Gene sets can be used individually, as a collection, or compared across collections in a number of ways including network analysis, genome-wide model representations, hierarchical clustering, gene set analysis (GSA) of microarray data, and principal component analysis (PCA) of gene set values; among others. A property of this collection of gene sets is that they describe complex intermediate and end stage phenotypes as opposed to molecular function or lists of coordinately regulated genes. They can be used in a variety of bioinformatics applications to reveal higher order or emergent biological and phenotypic relationships and to provide insight into the biological relevance of complex molecular datasets.

### Network analysis

**Figure 1:**
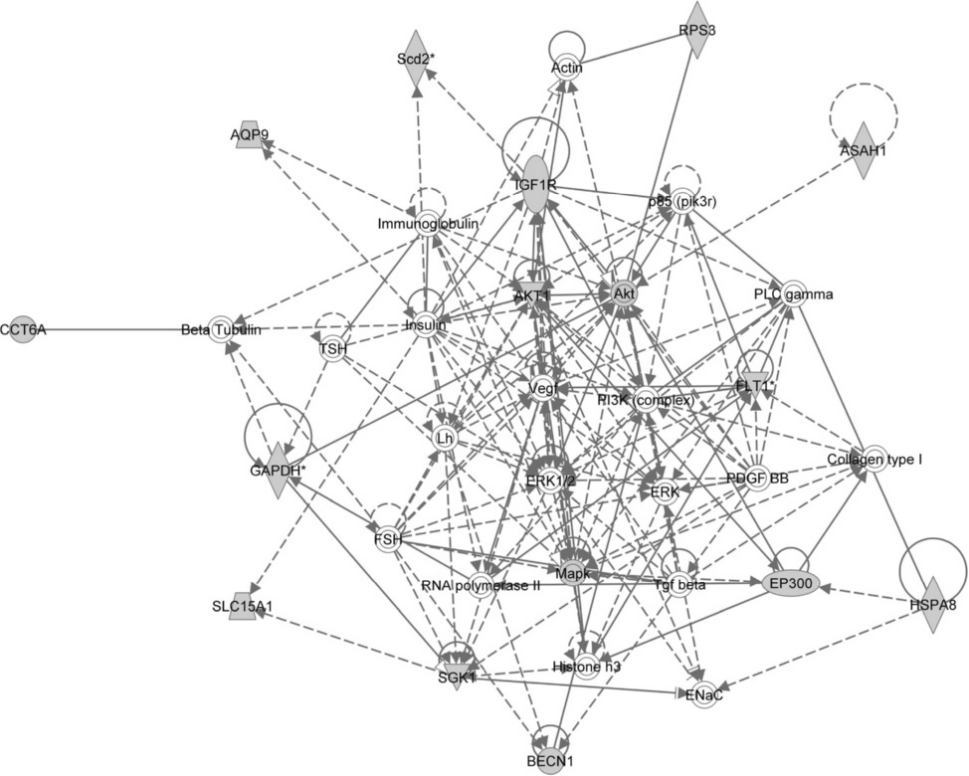
**Network Diagram of the single *C. elegans *gene set “life_span_variant” as produced by Ingenuity Pathway Analysis (IPA) shows members of the geneset (shaded) as well as regulatory or transcriptional partners not found (unshaded) in the original geneset.** The network highlights the central role of insulin, ERK family members, and PI3 Kinase as important contributors to longevity in worms.

Each individual gene set can be used to build networks to determine transcriptional regulation or protein-protein interactions. Figure 1 is a representative network of six networks showing regulatory relationships analyzed by Ingenuity Pathway Analysis (IPA) (Ingenuity® Systems, http://www.ingenuity.com) from a single 169 gene, *C*. *elegans* gene set, “life span variant”, found in the worm CE-Combined-GS 7-28-2011 file. This analysis identifies members of the gene set (shaded) as well as regulatory or transcriptional partners not found (unshaded) in the original gene set. This network highlights the central role of insulin, ERK family members, and PI3 Kinase as important contributors to longevity in worms.

**Figure 2:**
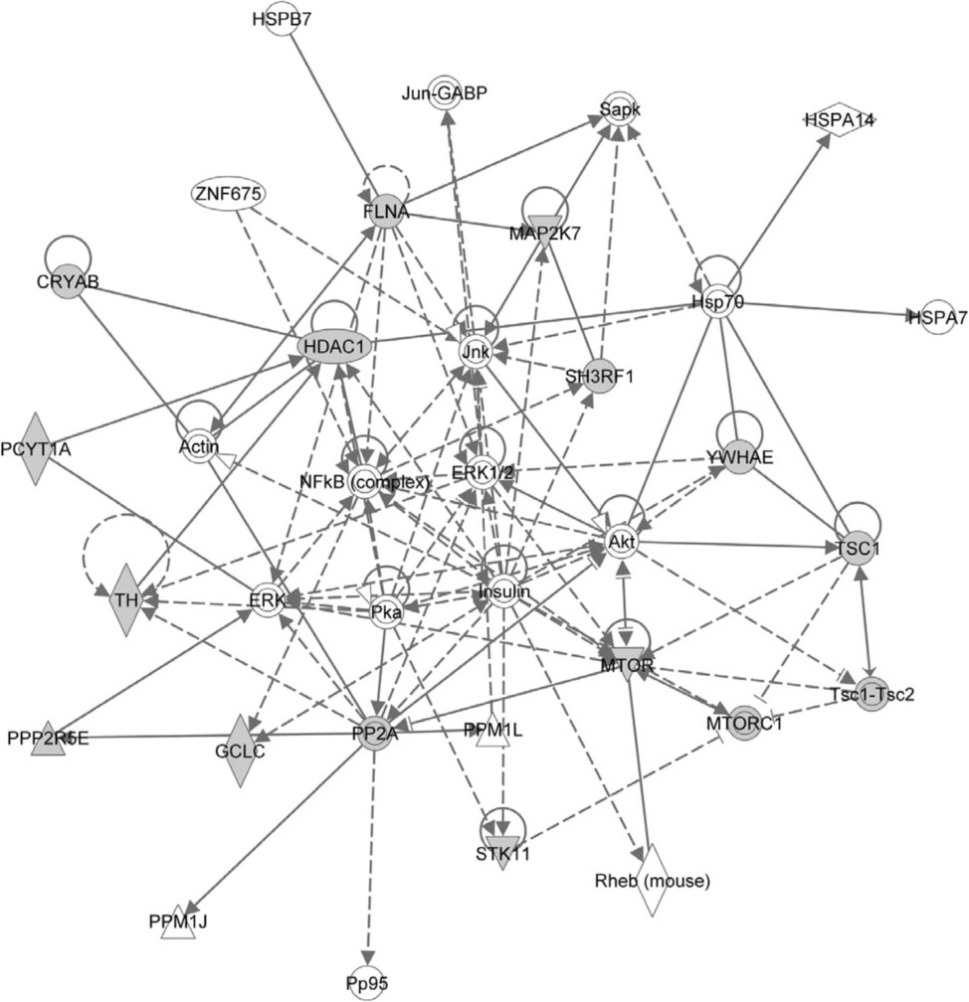
**Network Diagram of the single *D. melanogaster *gene set “long_lived” as produced by Ingenuity Pathway Analysis (IPA) as in the worm, insulin is central in this fly network, as well as ERKs, AKT, and histones, demonstrating significant overlap in age related biochemical pathways between worms and flies.**

An example of a network showing regulatory relationships from a single 82 gene “long_lived” gene set, found in the fly gene set file (DM-narrow-GS 9-7-2011), is also shown in Figure 2. Like in the worm, insulin is central in this fly network, as well as ERKs, AKT, and histones, demonstrating significant overlap in age related biochemical pathways between worms and flies. Each individual gene set (one phenotype with one row of annotated genes) produces multiple network diagrams showing the transcriptional neighbors and protein-protein partners of the core genes, while the entire collection of thousands of gene sets would produce many thousands of individual networks relative to phenotypic descriptions.

### Genome-wide phenotypic modeling in worms and flies

**Figure 3:**
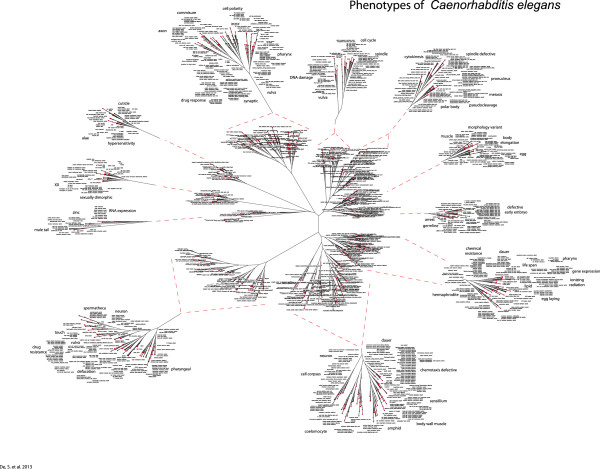
**Genome-wide modeling of 931 *C. elegans *gene sets, each with a minimum of 3 genes shows eleven large branches enriched for related biological functions.** Local relationships within a specific branch suggest functional relationships between closely spaced individual gene lists.

In addition to analysis of a single gene set, a collection of phenotypic gene sets can be compared to itself to reveal biological relationships between all members of the collection. Figure 3 shows a dendrogram of the combined *C*. *elegans* file (CE-Combined-GS), using gene sets, having three or more genes, compared to each other based on the degree of gene sharing between individual gene sets. The overall worm tree (Figure 3) is composed of eleven large branches enriched for related biological functions. Moreover, local relationships within a specific branch suggest functional relationships between closely spaced individual gene lists. For instance, in branch 2 (Additional file 5: Figure S1) cell cycle phenotypes such as “cell cycle timing”, “cell cycle delayed” and “cell cycle variant” are closely positioned in space and close to spindle assembly phenotypes. Likewise, in branch 6 (Additional file 6: Figure S2) Dauer phenotypes are closely aligned with multiple lifespan phenotypes based on individual gene sharing within their respective gene sets. Close apposition of related phenotypes as determined by gene sharing between gene sets is a pervasive feature of these dendrogram displays and represents overlap of related phenotypes being influenced by shared genes.

**Figure 4:**
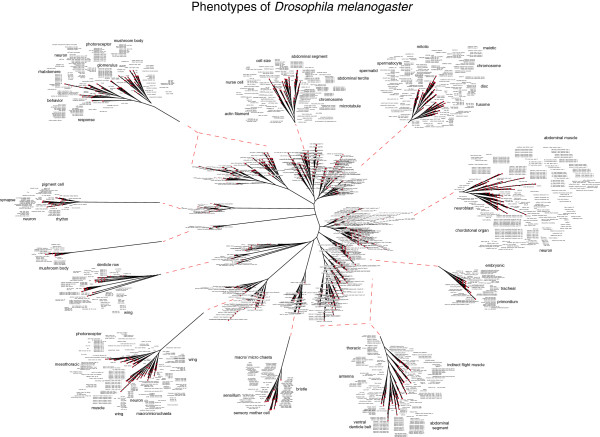
**Genome-wide modeling of 1,503 *D. melanogaster *gene sets, each with a minimum of 5 genes showing broad phenotypic groups represented by branches and a functional relatedness within subregions in each branch.**

The *Drosophila* gene set collection also produced a similar complex dendrogram of phenotypic functional groups based on gene sharing between gene sets (Figure 4). Like the worm dendrogram, individual branches of the fly dendrogram display a functional relatedness within subregions in each branch. For example, chromosome related phenotypes are grouped in branch 2 (Additional file 7: Figure S3) with mitotic and meiotic phenotypes, including meiotic telophase phenotypes, being closely aligned to each other, as well as spermatid and spermatocyte phenotypes. Behavioral, neuronal, and sensory response phenotypes are shown closely aligned in branch 11 of Figure 4 (Additional file 8: Figure S4), demonstrating overlapping genetic control of related complex phenotypes.

### Phenotype Gene Set Analysis (GSA) of microarray data and Principal Components Analysis (PCA) of gene sets

**Figure 5:**
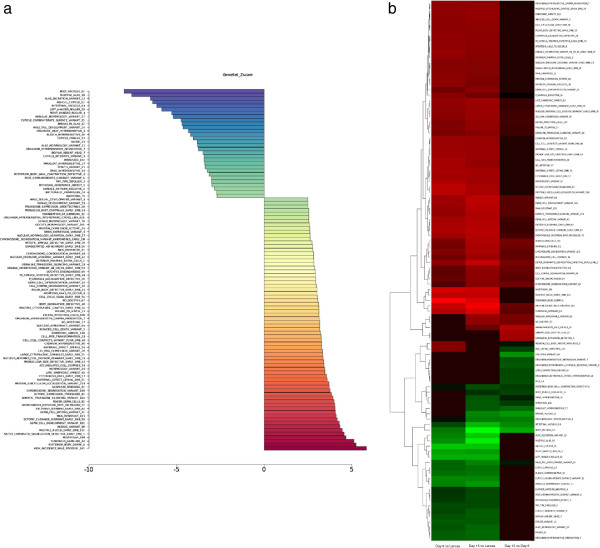
**a Phenotype gene set analysis of whole genome microarray expression values from 4 day larva versus 15 day *C. elegans *(GEO# GSE21784).** This was performed using the combined *C*. *elegans* gene set file on the Disease/Phenotype WEB-PAGE GSA web tool found here: *http://dpwebpage.nia.nih.gov/* which shows age-related changes in the worm. **b Heatmap analysis of whole genome microarray expression values from 4 day larva to 15 day *C. elegans *(GEO# GSE21784).** This was performed using the combined *C*. *elegans* gene set file on the Disease/Phenotype WEB-PAGE GSA web found here: *http://dpwebpage.nia.nih.gov/* which shows age-related changes in the worm.

*C*. *elegans*: In addition to comparisons of gene sets either individually or collectively to themselves, these phenotype gene sets are useful in analysis of microarray based gene expression datasets in worm and fly. Figure 5a illustrates statistically significant gene sets resulting from Gene Set Analysis (GSA) of a single 4 day old larva versus 15 day old whole genome gene expression comparison in a *C*. *elegans* aging microarray dataset [15]. This dataset (GEO # GSE21784) represents a 15 day time course with incremental stages of infection with *P*. *areuginosa*. Statistically significant up-regulated gene sets include germ cell gene groups, as well as meiosis and cell division gene sets, among others. Down-regulated gene sets include gene groups involved in body vacuoles, as well as alae and cuticle formation. Figure 5b is a heat map of the significant changes across the entire time course.

**Figure 6:**
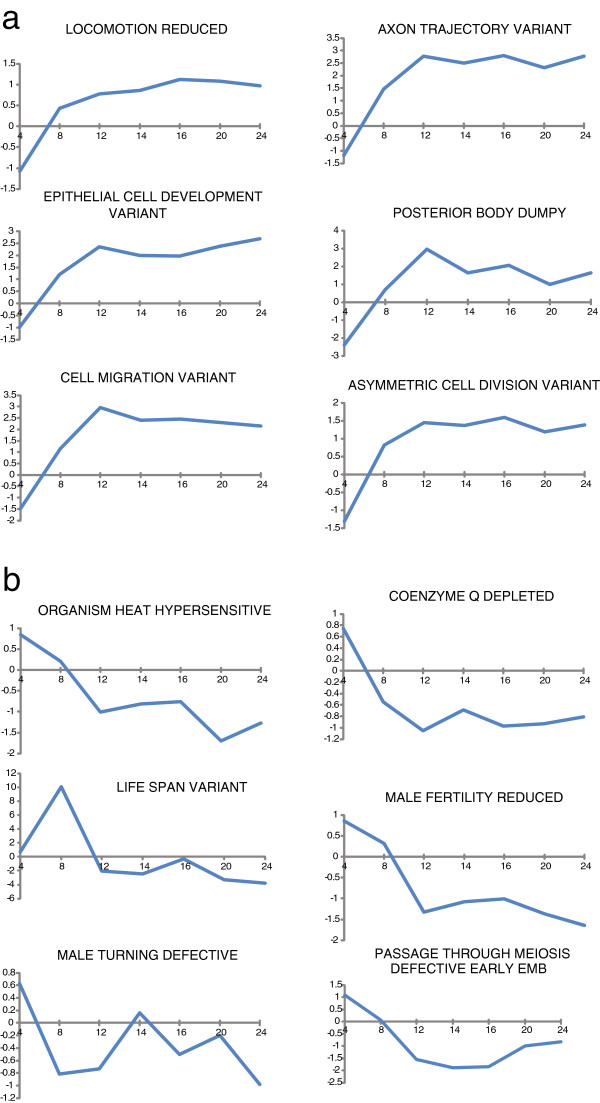
**Selected changes in gene sets over a *C. elegans *aging time course.** (GEO# GSE12290) showing increases **(a)** and decreases **(b)** in gene set scores over time. The X axis shows days 4,8,12,14,16,20,24 and the X axis shows Z-score for each gene set shown.

Figure 6 shows changes in selected gene sets from a different aging time course in *C*. *elegans* over 24 days [16] (GEO # GSE12290). Aging related increases (Figure 6a) or decreases (Figure 6b) in gene groups related to locomotion, energy metabolism, and life span are highlighted.

**Figure 7:**
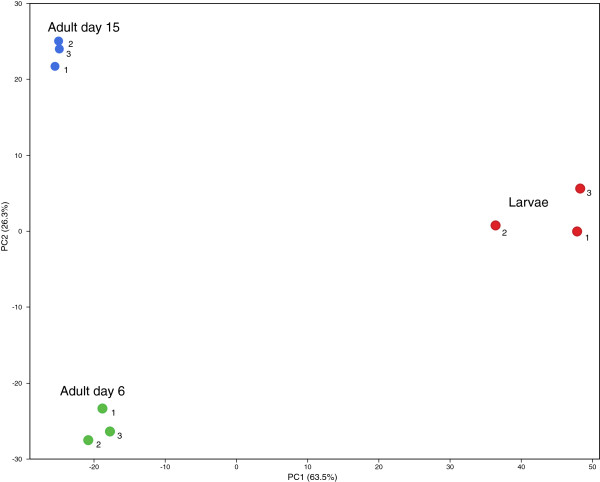
**Two dimensional Principal Components Analysis (PCA) of gene set results from whole genome microarray expression values from *C. elegans *larva, 6 day adult worms, and 15 day Adult *C. elegans *(GEO# GSE21784) showing age as the principal variation component.**

In addition to GSA of microarray data the gene set values derived from gene expression data can be further analyzed by principal components analysis (PCA) using the Z-score values of the original gene set data output. This is in contrast to more commonly described PCA resulting from individual gene expression values. Figure 7 shows tight grouping of individual biological samples within three groups; larvae, adult day 6, and adult day 15, and dramatic separation of time points within the experiment, based solely on PCA analysis of the gene sets values from the previous gene set analysis. This demonstrates that there is useful biological information content in the aggregate gene set results, in addition to that found in any individual gene set, which can discriminate between discrete biological states.

**Figure 8:**
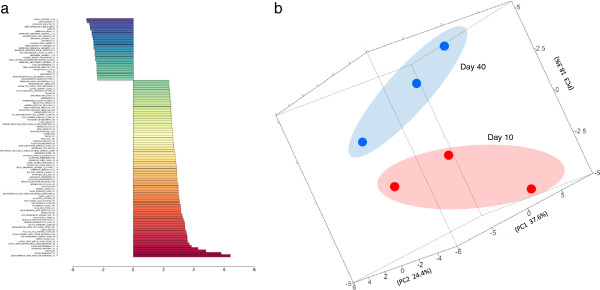
**a Phenotype gene set analysis of average whole genome microarray expression values from 10 day versus 40 day *D. melanogaster *heads (GEO# GSE22437).** This was performed using the *D*. *melanogaster* gene set file on the WEB-PAGE GSA web tool showing age-related changes in the fly. **b Principal Components Analysis of gene set results from of whole genome microarray.** Gene expression values from *D. melanogaster* 10 day and 40 day *Drosophila* heads (GEO# GSE22437) showing age as the principal variation component.

*D*. *melanogaster*: In a similar fashion to the worm (above), microarray data from young versus aging flies was analyzed with the *Drosophila* gene set file DM-narrow-GS containing 11,999 gene sets. Gene set analysis was performed using the WEB-*PAGE* gene set analysis tool [10] on a dataset of gene expression values from young versus old flies [17] (GEO# GSE22437). The top 100 statistically significant enriched gene sets using Z ratios of the expression values from day 10 versus day 40 fly heads is shown in Figure 8. Over enriched gene sets include minute phenotypes, life span, as well as developmental growth rate phenotypes, among others. The discriminative ability of PCA using gene set Z-scores (as opposed to individual gene values) is illustrated using the individual samples of day 10 versus day 40 fly heads in Figure 8.

## Conclusion

Here we describe genome-wide phenotypic modeling using gene sets based on gene-phenotypic assignments in *C*. *elegans* and *D*. *melanogaster*. Unlike previous gene set collections such as KEGG, GO, MSigDB, in these and other species, every gene in every gene set described here is based on genetic evidence contributing to each specific phenotype. Although very useful, these gene sets should be considered a first generation. They may not be complete. Some may describe certain phenotypes in different developmental contexts, or in particular applications and not in others. In addition, many subtleties and details were not included in deriving these gene sets including penetrance of different alleles, strain differences, and environmental modifiers. Moreover, these gene sets may produce different results depending on the statistical algorithms used in complex analysis.

However, we have demonstrated these gene sets can be used to identify complex higher order biological and genetic relationships through network analysis, whole genome phenotypic modeling, and analysis of complex molecular datasets. They will help elucidate complex multigenic relationships between genes and phenotypes in worms and flies in many experimental and biological contexts and will provide a bridge for phenotypic comparisons between model and intermediate species.

## Methods

### Derivation of phenotypic gene sets

#### Worm

Phenotype-gene lists obtained from WormBase on 4/24/11 were titled RNAi and VAR. RNAi, consisted of 34,433 gene phenotype pairs having 7,289 unique genes and 850 unique phenotypes. These phenotypes were the results of observations of phenotypes from knockdown of the gene products (RNAi experiments). The list VAR contained 8,440 records, having 2,165 unique genes, and 1,109 unique phenotypes and was the result of observations of phenotypes from genetic mutations as deposited in WormBase. The overlap between each file consists of 1,410 genes and 237 phenotypes.

Phenotype gene set files were created by parsing the original gene lists into non-redundant phenotype lists with annotated genes using a custom Perl script as previously described [9]. This was done for RNAi and VAR independently, as well as combined to create the gene set files; CE-RNAi-GS 7-26-11, CE-VAR-GS 7-26-11, and CE-Combined-GS 7-28-11. The resultant individual Phenotype Gene set names are identical to the Phenotype descriptors found in the original WormBase Phenotype file. These files can be downloaded here: http://www.grc.nia.nih.gov/branches/rrb/dna/index/Worm-fly_gene_sets_5-9-12.html.

#### Fly

Phenotypes and gene assignments were obtained from FlyBase on 9-11-11 at this web address: http://FlyBase.org/static_pages/downloads/FB2011_07/alleles/allele_phenotypic_data_fb_2011_07.tsv.gz. This file began with 259,162 phenotypic descriptions with assigned *Drosophila* genes. Redundant phenotype-gene combinations were removed resulting in a list of 154,428 unique phenotype-single gene pairs. Parsing of this file resulted in a non-redundant gene set file of 11,999 unique phenotypic terms with annotated genes. The resultant individual Phenotype Gene set names are identical to the Phenotype descriptors found in the original FlyBase Phenotype file. This Phenotype gene set file named DM-narrow-GS 9-7-2011, can be downloaded here: http://www.grc.nia.nih.gov/branches/rrb/dna/data/worm-fly/DM-narrow-GS_9-7-2011.txt.

### Gene set nomenclature

It should be noted that nomenclature of many phenotype gene sets in both worm and fly often have a directionality in the name which may or may not be relevant to any given microarray or other analysis. Please see Additional file 9: S7 for an explanation of directionality in gene set nomenclature and interpretation in their use.

### Network analysis

Networks for *C*. *elegans* and *D*. *melanogaster* were produced using Ingenuity Pathway Analysis (IPA) (Ingenuity® Systems, http://www.ingenuity.com). Using the “life_span_variant” gene set in *C*. *elegans* generated on 7-26-2011, and the “long_lived” gene set in *D*. *melanogaster* generated on 12-07-2011. The input and output files can be found here for *C*. *elegans* (Additional file 10: Table S5) and *D*. *melanogaster* (Additional file 11: Table S6).

### Genome-wide phenotypic modeling

Genome-wide dendrograms were produced by a unique method similar to phylogenetic classification as previously described [9]. Briefly, the distance between each phenotypic gene set was calculated by pairwise comparison of every gene set pair by finding the number of common genes between each pair and dividing that number by the number of genes in the smallest group of the pair, resulting in a correlation value between 1 and 0 for each pair. This was done for all gene sets to produce a distance matrix. This number was then subtracted from 1because if two lists are identical (100 % match) then the resultant distance should be 0. This is represented as:

$$d_{i,j}=1-\frac{N\left(C_{i}\cap C_{j}\right)}{\min\left[N\left(C_{i}\right),N\left(C_{j}\right)\right]}$$

*when i* ≠ *j*. If *i* = *j* then d = 0

Where: *C*_*k*_: Genes in each disease set (where *k* = *i*,*j*) ; N(*C*_*k*_): Number of genes in each disease set (where *k* = *i*,*j*) ; d_ij_ is the pairwise distance ; *i*,*j*: index of genes in each disease set where; *i* = *1*,*2*,*3*,………,*n* ; *j* = *1*,*2*,*3*,………,*m*.

The gene set relationships were calculated from the distance matrix using the Fitch program [18]. It calculates the relationships based on the Fitch and Margoliash method of constructing the phylogenetic trees using the following formula (from the Phylip manual):

$$\mathrm{Sum}\_\mathrm{of}\_\mathrm{squares}=\sum_{i}\sum_{j}\frac{n_{\mathrm{ij}}{\left(D_{\mathrm{ij}}-d_{\mathrm{ij}}\right)}^{2}}{D_{\mathrm{ij}}{}^{P}}$$

where *D* is the observed distance between gene sets *i* and *j* and *d* is the expected distance, computed as the sum of the lengths of the segments of the tree from gene set *i* to gene set *j*. The quantity *n* is the number of times each distance has been replicated. In simple cases *n* is taken to be one. If *n* is chosen more than 1, the distance is then assumed to be a mean of those replicates. The power *P* is what distinguished between the Fitch and Neighbor-Joining methods. For the Fitch- Margoliash method P is 2.0 and for Neighbor-Joining method it is 0.0. The resulting coefficient matrix file was displayed using the Phylodraw graphics program [19].

### Gene set analysis

This analysis used the Disease/Phenotype WEB-*PAGE* GSA web tool[10] using the PAGE algorithm [2] with the CE-Combined-GS gene set file excluding gene sets containing over 500 and less than 3 genes. Briefly, for each gene set a Z score was computed as, $Z_{\mathrm{phenotype}}\left(i\right)=\frac{\sqrt{n_{i}-1}•\mathrm{dif}f_{i}}{\sigma_{a}}$ In which the phenotype index *i* = 1,2,…,K; where K is the total number of the disease phenotypes we included in our data set; n_I_ is the number of genes in the sub-group of phenotype *i* in the current sample array; σ_A_ is the standard deviation of the current gene expression changes of the sample. Diff(*i*): is the difference between the mean value of gene expression changes in the subgroup disease phenotype (*i*) (GC_I_ ) and the mean value of the gene expression changes on the whole sample (GC_A_) i.e. $\mathrm{dif}f_{i}=\bar{GC_{i}}-{\bar{GC}}_{a}$. The empirical p-value of the disease phenotype *i* changes is described by: $p\left(i\right)=2\left[1-\Phi \left(\frac{\mathrm{dif}f_{i}}{\sigma \left(\mathrm{dif}f_{i}\right)}\right)\right]$ in which Φ(x) is the standard normal distribution function with the variable as X = DIFF_I_/σ(DIFFF_I_). σ(DIFF_I_) is the standard deviation of the difference for gene expression changes between phenotype subgroup (*i*) and the whole array $\sigma \left(\mathrm{dif}f_{i}\right)=\sqrt{\frac{\sigma_{i}{}^{2}}{n_{i}}+\frac{\sigma_{a}{}^{2}}{n_{a}}}$ σ_I_ is the standard deviation of the average gene expression changes in the disease phenotype (*i*). N_A_ is the total number of genes in the whole sample set. The plots were drawn with R-statistical programming language (R Development Core Team 2005) using either calculated or absolute z-score values.

### Principal components analysis

Principal components analysis was performed on the gene set Z values using DIANE 8.0 a JMP based software package (http://www.grc.nia.nih.gov/branches/rrb/dna/diane_software.pdf) based on the Singular Value Decomposition (SVD) function in JMP 9.0. In short, the data was organized as m × n matrix where m is the different samples (columns) and n is gene set Z-values (rows), mean of each row was subtracted and SVD was calculated using JMP’s in-built SVD function as illustrated in this document: http://www.cs.princeton.edu/picasso/mats/PCA-Tutorial-Intuition_jp.pdf and also used in this script: http://abs.cit.nih.gov/MSCLtoolbox.

### Data access

The complete *C*. *elegans* and *D*. *melanogaster* gene set files are available at this address: http://www.grc.nia.nih.gov/branches/rrb/dna/index/Worm-fly_gene_sets_5-9-12.html.

## Competing interests

The authors declare that they have no competing interests.

## Authors’ contributions

SD participated in study design and implemented the graphing algorithm. YZ participated in study design and developed the primary gene set files for both species. CW, SZ, and IG provided biological insights into the relevance and applicability in both C. elegans and D melanogaster. KGB conceived the study design, participated in gene set development, ran analysis, and wrote the manuscript. All authors read and approved the final manuscript.

## Supplementary Material

Additional file 1: Table S1 The complete phenotype gene sets for *C*. *elegans*. Click here for file

Additional file 2: Table S2 The RNAi phenotype gene sets for *C*. *elegans*. Click here for file

Additional file 3: Table S3 The VAR phenotype gene sets for *C*. *elegans*. Click here for file

Additional file 4: Table S4 The complete phenotype gene sets for *D*. *melanogaster*. Click here for file

Additional file 5: Figure S1 Branch 2 of the gene set dendrogram of *C*. *elegans*. Click here for file

Additional file 6: Figure S2 Branch 6 of the gene set dendrogram of *C*. *elegans*. Click here for file

Additional file 7: Figure S3 Branch 2 of the gene set dendrogram of *D*. *melanogaster*. Click here for file

Additional file 8: Figure S4 Branch 11 of the gene set dendrogram of *D*. *melanogaster*. Click here for file

Additional file 9: S7 Directionality in gene set nomenclature and interpretation in their use. Click here for file

Additional file 10: Table S5 The gene set for *C*. *elegans* for “life_span_variant”. Click here for file

Additional file 11: Table S6 The gene set for *D*. *melanogaster* for “long lived”. Click here for file
