# Supplementary figures and images for: Genome-wide modeling of complex phenotypes in Caenorhabditis elegans and Drosophila melanogaster
Source: BMC Genomics. 2013 Aug 28;14:580. doi: 10.1186/1471-2164-14-580 (PMC3849582; doi:10.1186/1471-2164-14-580)

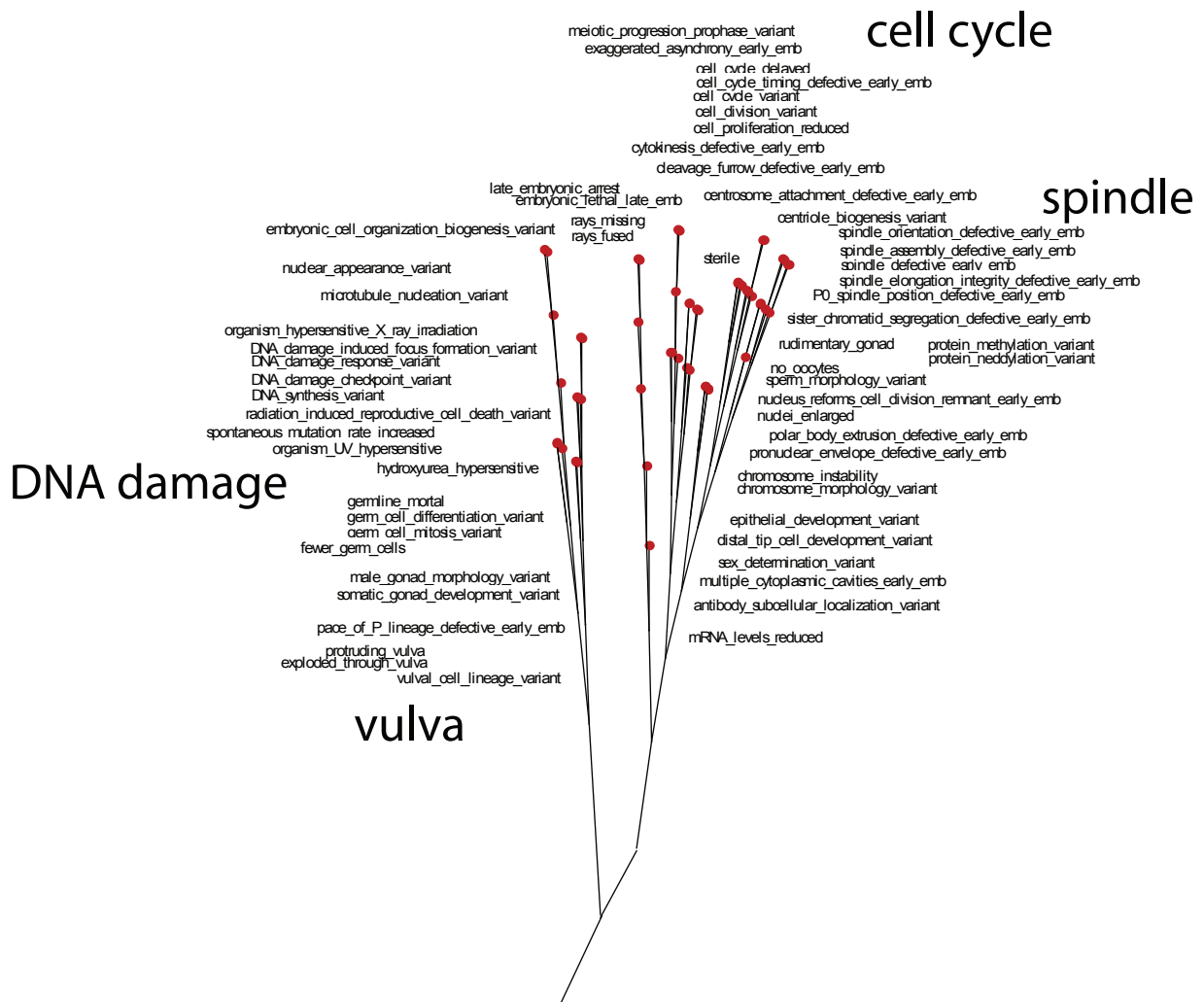

Supplemental Figure 1  
Branch 2 of the gene set dendrogram of *C. elegans*

Supplement: Additional file 5: Figure S1 — Branch 2 of the gene set dendrogram of C. elegans. [file 1471-2164-14-580-S5.pdf]

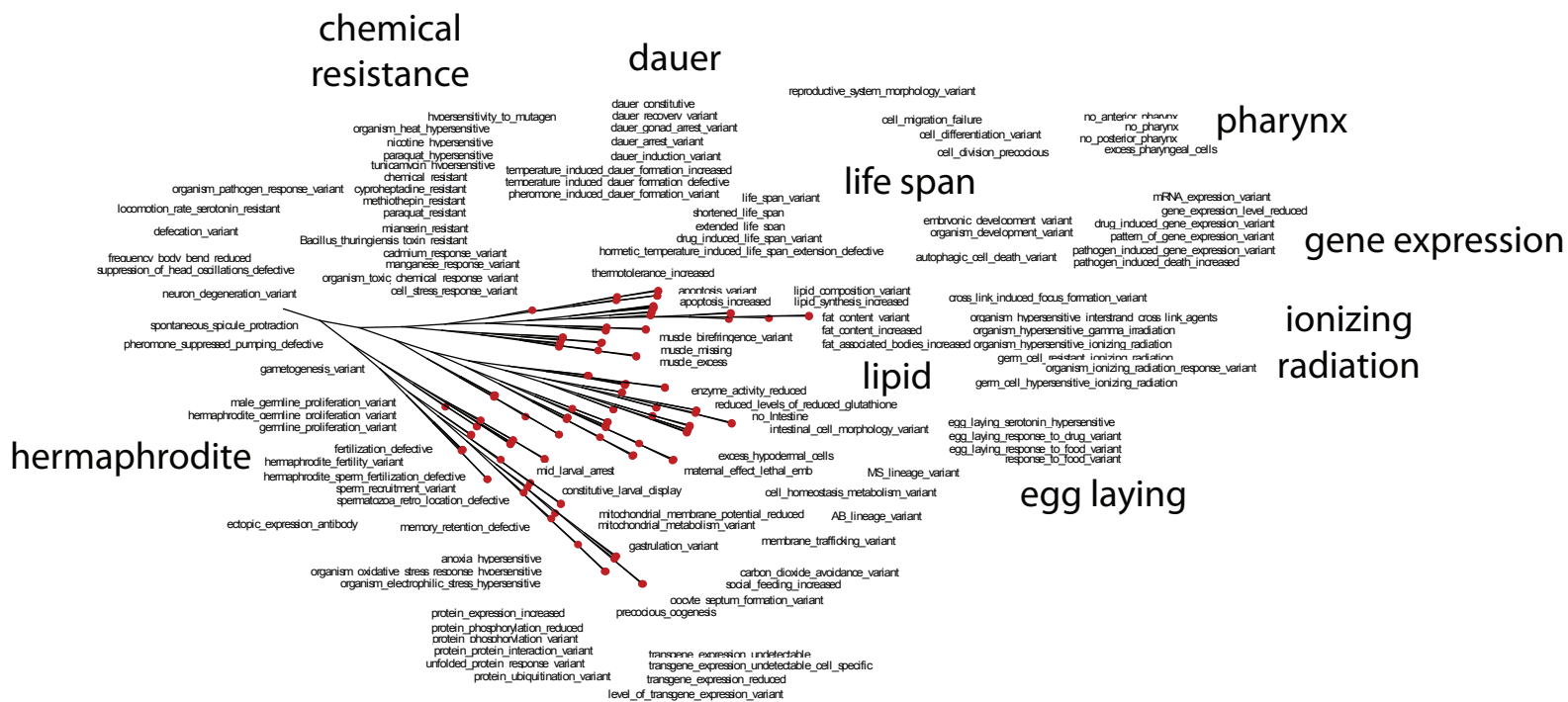

Supplemental Figure 2  
Branch 6 of the gene set dendrogram of *C. elegans*

Supplement: Additional file 6: Figure S2 — Branch 6 of the gene set dendrogram of C. elegans. [file 1471-2164-14-580-S6.pdf]
