# Supplementary material for: Genome-wide modeling of complex phenotypes in Caenorhabditis elegans and Drosophila melanogaster
Source: BMC Genomics. 2013 Aug 28;14:580. doi: 10.1186/1471-2164-14-580 (PMC3849582; doi:10.1186/1471-2164-14-580)
Supplement: Additional file 7: Figure S3 — Branch 2 of the gene set dendrogram of D. melanogaster. [file 1471-2164-14-580-S7.pdf]

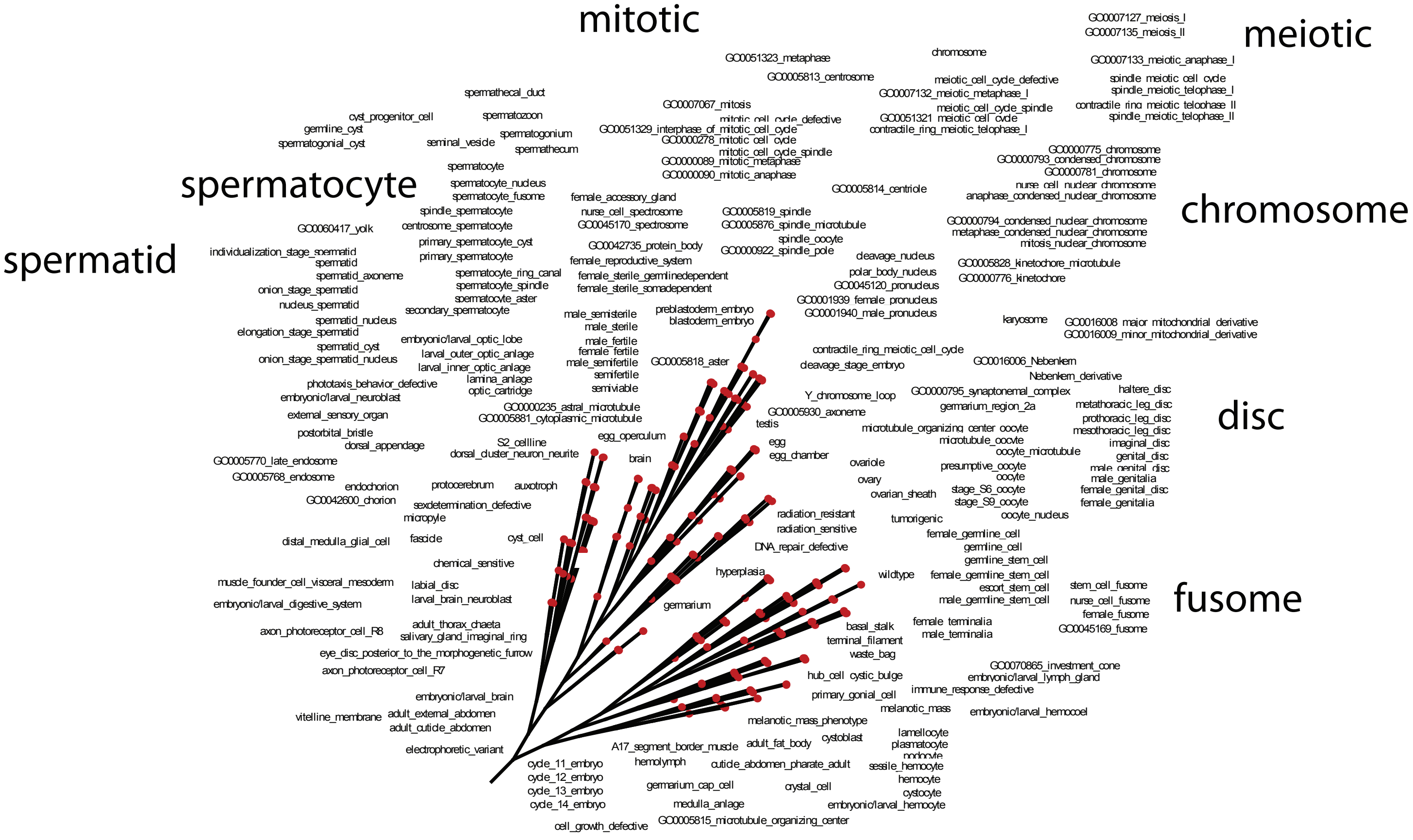

Supplemental Figure 3
Branch 2 of the gene set dendrogram of *D. melanogaster*
