# Supplementary material for: Genome-wide modeling of complex phenotypes in Caenorhabditis elegans and Drosophila melanogaster
Source: BMC Genomics. 2013 Aug 28;14:580. doi: 10.1186/1471-2164-14-580 (PMC3849582; doi:10.1186/1471-2164-14-580)
Supplement: Additional file 8: Figure S4 — Branch 11 of the gene set dendrogram of D. melanogaster. [file 1471-2164-14-580-S8.pdf]

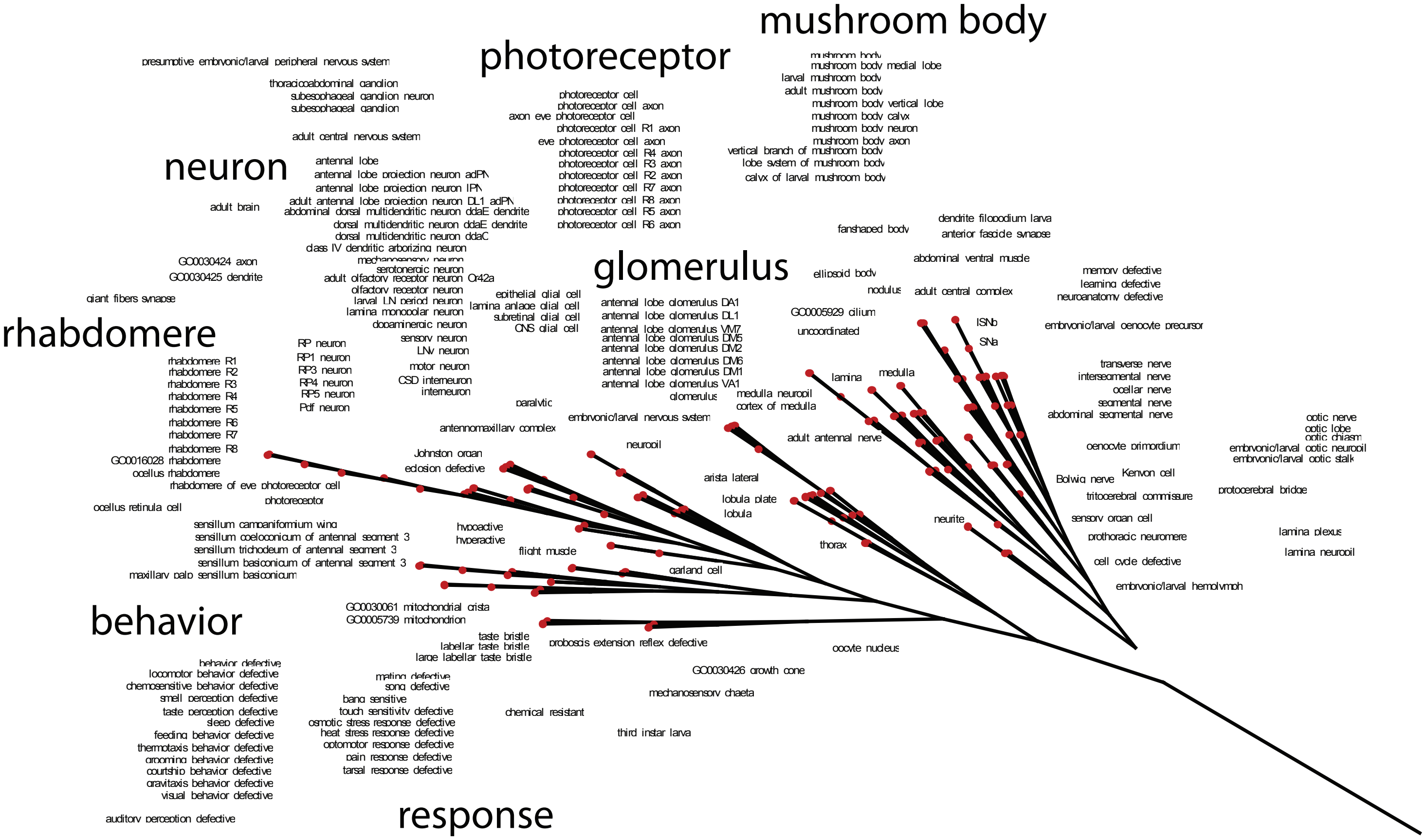

Supplemental Figure 4
Branch 11 of the gene set dendrogram of *D. melanogaster*
